# Subjective Social Status and Cardiovascular Reactivity: An Experimental Examination

**DOI:** 10.3389/fpsyg.2016.01091

**Published:** 2016-07-19

**Authors:** Karoline Pieritz, Philipp Süssenbach, Winfried Rief, Frank Euteneuer

**Affiliations:** ^1^Division of Clinical Psychology and Psychotherapy, Philipps UniversityMarburg, Germany; ^2^Division of Psychological Methods, Philipps UniversityMarburg, Germany

**Keywords:** subjective social status, cardiovascular reactivity, blood pressure, heart rate variability, cardiovascular health

## Abstract

The present experiment examined the causal influence of subjective social status (SSS) on variables related to cardiovascular health [i.e., blood pressure, heart rate variability (HRV)]. Participants were randomly assigned to one of two conditions involving a social comparison that either induced a temporary shift toward high SSS or toward low SSS. Cardiovascular variables were measured before (baseline), throughout, and after the manipulation (recovery). Participants in the low SSS condition had a significantly lower HRV during experimental manipulation than at baseline (*p* = 0.001). They also showed a significantly stronger HRV reactivity compared to participants in the high SSS condition (*p* = 0.027). Our results suggest that already temporary shifts of one's SSS have measureable effects on cardiovascular variables. They support the notion that social status plays a causal role in the development of cardiovascular disease.

## Introduction

Lower social status has been associated with a wide range of negative health outcomes including an increased risk for cardiovascular diseases (CVD) (Adler and Ostrove, 1999; Clark et al., 2009; Euteneuer, 2014), the most common causes of death globally (World Health Organisation, 2014). In addition to traditional measures of objective social status (OSS) such as education, income, and occupation, research has increasingly focused on the link between subjective social status (SSS) and health. SSS refers to an individual's perceived social position relative to other members in their social environment. As such it is not only determined by objective characteristics but also by people's general life satisfaction, the way they perceive their position in comparison with relevant others, and what they assume their position will be in future (Cundiff et al., 2013).

Research suggests that the relationship between health and SSS is stronger than the relationship between health and OSS (Euteneuer, 2014). One explanation for the tighter relationship between SSS and health is that SSS provides a more comprehensive measure of one's social position—possibly by enabling a cognitive averaging of a broader range of status-related information, taking into account the social position *relative* to other members in one's social environment (Singh-Manoux et al., 2005). Cross-sectional and longitudinal studies found associations between lower SSS and poorer cardiovascular health, lower self-rated health, mental disorders including depression, higher substance use, diabetes, and higher mortality (Euteneuer, 2014). Moreover, SSS is related to various stress-related biological risk factors for disease as well as alterations in the endocrine, immune, cardiovascular, and autonomic nervous systems such as sympathetic overactivation, increased resting heart rate and blood pressure, higher BMI, altered cortisol responses, and decreased immune functioning which might be relevant for the pathophysiology of CVD (Euteneuer, 2014).

Importantly, the concept of SSS involves a situational component as it reflects a person's perception of his or her status *relative* to others. Thus, while SSS is predicted by indicators of OSS that are relatively stable, it is also based on a social comparison with others. This comparison process provides an opportunity to experimentally manipulate an individual's SSS. On the basis of paradigms for cultural identity (Oyserman and Lee, 2008) and the MacArthur Scales (Cohen, 1999; Adler et al., 2000). Kraus et al. (2010) developed a paradigm for a temporal manipulation of SSS in which participants are instructed to compare themselves with people at the very top or at the very bottom of society. This comparison results in a contrast effect leading to temporarily reduced SSS vs. elevated SSS, respectively. Studies applying this paradigm yielded promising results. They found that temporary changes in SSS affect social behavior such as empathic accuracy (Kraus et al., 2010), unethical behavior (Piff et al., 2012), or charitable donations (Piff et al., 2010).

To our knowledge, no study has yet examined the impact of a temporary shift in SSS on physiological processes. With respect to the strong associations of SSS and cardiovascular health factors in cross-sectional and longitudinal studies, the present study aims to extend the existing findings on SSS and cardiovascular processes by examining whether an experimental manipulation of SSS affects physiological processes relevant for the pathophysiology of CVD such as blood pressure regulation and heart rate variability (HRV). We hypothesize that a social comparison with high-status individuals (low SSS condition) elicits stronger cardiovascular reactivity than a social comparison with low-status individuals (high SSS condition).

## Methods

### Participants

Assuming an effect size of *d* = 0.54 (for the effect of the social class manipulation on SSS as reported by Kraus et al., 2010), we strived to collect data from 66 participants for a power of 0.70. Two participants were excluded from the analyses, one because he didn't understand the task and one because of medication intake. The remaining participants were 64 healthy university students (mean age 24.3 ± 3.9 years). They were recruited via email announcements and university bulletin boards. Telephone screenings were conducted using a standardized checklist to control for the following exclusion criteria: chronic illness, health problems, and medication intake as well as mental health conditions which may affect attention and/or cardiovascular processes. The study was approved by a local ethics committee. All subjects gave written informed consent in accordance with the Declaration of Helsinki.

### Experimental manipulation of subjective social status (SSS)

The experimental manipulation of SSS relied on a paradigm developed by Kraus et al. (2010) and used the German version of the MacArthur Scales to measure the success of the manipulation (Euteneuer et al., 2015). Participants were presented with an image of a ladder with 10 rungs. They were instructed to think of the ladder “as representing where people stand in Germany.” They were then randomly assigned to one of two conditions in which they were instructed to compare themselves with someone who has a higher social status (low SSS condition) or a lower social status (high SSS condition):

“On the very bottom [top] of the ladder are people who are the worst [best] off—those who have the least [most] money, least [most] education, and the least [most] respected jobs. Now, please compare yourself to the people on the very bottom [top] of the ladder. We'd like you to think about how you are different from these people in terms of your own income, educational history, and job status and how you feel disadvantaged [advantaged] compared to them. Where would you place yourself on this ladder relative to these people at the very bottom [top]?”

To strengthen the manipulation, we instructed participants to talk loudly about these differences for 5 min. Participants then indicated their own standing on the ladder; the bottom rung was coded as “1,” and the top rung was coded as “10.”

In the case of a successful experimental manipulation, one would expect that participants in the low SSS condition would place themselves significantly lower on the ladder than participants with a high SSS condition.

### Cardiovascular measures

Blood pressure (BP), heart rate (HR), and HRV were obtained using a Task Force Monitor^®;^ 3040i device (TFM, CNSystems, Graz, Austria). The TFM application allows an automated and computed beat-to-beat analysis of HR [electrocardiogram (ECG)] using oscillometric and noninvasive continuous blood pressure measurements. Hemodynamic and autonomic parameters are calculated on the basis of these signals. The TFM offers valid and reliable measurements of all parameters and has been used successfully in recent clinical studies (Parati et al., 2003; Fortin et al., 2006). Mean, median, minimum, maximum, and standard deviation (SD) of all parameters were calculated automatically for three predefined 5 min intervals: resting interval before the beginning of the experiment (baseline), the experimental manipulation (experiment), and another resting interval after the experimental manipulation (recovery).

Systolic and diastolic BP were analyzed separately. For further analyses of HRV, two of the most widely used time domain indices, the standard deviation of normal-to-normal intervals (SDNN), and the square root of the mean squared differences of successive normal-to-normal intervals (RMSSD) were chosen (Rajendra Acharya et al., 2006). We chose time domain variables because these are equivalent to frequency-domain variables and are easier to perform (Task Force of The European Society of Cardiology and The North American, and Society of Pacing and Electrophysiology, 1996). SDNN is a good predictor of overall variability present at the time of recording. It reflects long-term variability of cardiac activity and is influenced by both sympathetic and parasympathetic activity (von Borell et al., 2007). RMSSD reflects short-term alterations of HRV and is considered to be predominantly an indicator of in parasympathetic tone. Despite being highly correlated to power spectral measures of respiratory sinus arrhythmia, it has been suggested that RMSSD is not significantly affected by changes in breathing rate (Penttilä et al., 2001).

### Objective social status

Since income is an important objective social determinant of health (Sapolsky, 2004), participants' average monthly household-income was assessed according to Winkler (Winkler and Stolzenberg, 2009). Scores range from 1 to 7. Lower scores indicate lower household net income.

### Procedure

All participants were tested individually. The examinations were conducted by a male experimenter. After signing an informed consent, participants completed sociodemographic and psychological questionnaires (~30 min) which was followed by a baseline assessment of physiological measures (5 min). Subsequently, SSS was experimentally manipulated. Physiological parameters were measured throughout the manipulation (5 min). Participants' verbal responses were digitally recorded in order to check for accuracy of their responses at a later time. Experimental manipulation of SSS was followed by a third assessment of physiological measures (recovery; 5 min). The study was completed with a thorough debriefing.

### Data analysis

Statistical analyses were carried out with IBM SPSS Statistics version 22.0 for Windows (Chicago, SPSS, Inc.). Pairwise comparisons were calculated with *T*-tests, Mann–Whitney *U*-tests or chi-square tests as appropriate. To test for differences in blood pressure and HRV, analysis of variance (ANOVA) for repeated measures using time (baseline, experiment, and recovery) as the repeated factor and group (high SSS or low SSS) as the between-group factor were calculated. Significant time × group interactions were followed with pairwise comparisons. Since household net income, sex, age, body mass index (BMI), and smoking status have been shown to be associated with cardiovascular processes (Omvik, 1996; Franklin et al., 1997; Franklin, 1999; Kuo et al., 1999; Fagard, 2001; Sapolsky, 2004; Faheem et al., 2010; Thayer et al., 2010), analyses were adjusted for these variables.

## Results

### Baseline measures

Baseline sample characteristics are shown in Table 1. Although global OSS scores did not differ between groups (low SSS: *M* = 7.7, *SD* = 1.9; high SSS: *M* = 7.5, *SD* = 1.4), participants in the low SSS group reported a significantly higher household net income than participants in the high SSS condition (*p* = 0.046). This difference was no significant restriction since it was in the opposite direction of our hypotheses. There were no other significant baseline differences between participants in the low SSS and high SSS group (*p* > 0.1) indicating successful randomization.

**Table 1:** **Baseline sample characteristics**.

	**High SSS (*n* = 33)**	**Low SSS (*n* = 31)**	***p*-values**
Age, years	23.9 (4.1)	24.8 (3.6)	0.396
Females, n (%)	19 (57.6)	19 (61.3)	0.762
Current smoker, n (%)	6 (18.2)	4 (12.9)	0.345
Body mass index, kg/m^2^	21.6 (2.2)	21.6 (2.2)	0.971
Household net income	1.0 (0.0)	1.7 (1.8)	0.046
**BLOOD PRESSURE, mmHg**			
Systolic	110.5 (8.8)	109.3 (8.1)	0.571
Diastolic	69.0 (7.2)	70.7 (8.9)	0.437
Heart rate, bpm	75.0 (11.5)	75.1 (11.2)	0.978
**HEART RATE VARIABILITY**			
RMSSD	62.5 (24.0)	69.1 (26.1)	0.326
SDNN	53.9 (23.9)	64.2 (25.7)	0.116

*Values shown as mean (SD). bpm, beats per minute; mmHg, millimeters of mercury; OSS, objective social status (mean scores equal: educational level, general qualification for university entrance; profession, student/trainee; household net income: higher SSS, ≤ 250 €; lower SSS, 1250–1750 €), RMSSD, square root of the mean squared differences of successive normal-to-normal intervals; SDNN, standard deviation of normal-to-normal intervals; SSS, subjective social status*.

### Manipulation check

As a manipulation check, ratings of SSS between the two experimental conditions were compared. Since the Kolmogorov–Smirnov test indicated non-normality, non-parametric Mann–Whitney *U*-Test was used. As expected, participants in the high SSS condition (Median = 7) placed themselves significantly higher on the ladder than participants in the low SSS condition (Median = 5), *U* = 310, *p* = 0.034. Thus, the manipulation successfully shifted participants' social status perception in the expected direction.

### The effect of SSS on cardiovascular processes

Table 2 shows cardiovascular measures before (baseline), during and after experimental manipulation (recovery). With respect to HRV, repeated measures ACNOVA revealed a significant time × group interaction for RMSSD, *F*_(2, 98)_ = 3.27, *p* = 0.042, $\eta_{\text{p}}^{2}$ = 0.063. This effect indicates that RMSSD at different time points differed between experimental conditions. Pairwise comparisons revealed that in the low SSS group, RMSSD during experimental manipulation was significantly lower than at baseline, *t*_(27)_ = 3.89, *p* = 0.001, and significantly lower than at recovery, *t*_(27)_ = −3.39, *p* = 0.002. There were no significant RMSSD differences between time points for the high SSS group (*p* > 0.1).

**Table 2:** **Cardiovascular measures at baseline, experimental manipulation, recovery, and change scores (delta)**.

	**High SSS (*n* = 33)**	**Low SSS (*n* = 31)**	***p*-values**
**BASELINE**			
Systolic blood pressure, mmHg	110.5 (8.8)	109.3 (8.1)	0.571
Diastolic blood pressure, mmHg	69.0 (7.2)	70.7 (8.9)	0.437
HRV, RMSSD	62.5 (24.0)	69.1 (26.1)	0.326
HRV, SDNN	53.9 (23.9)	64.2 (25.7)	0.116
**EXPERIMENT**			
Systolic blood pressure, mmHg	124.6 (10.4)	119.1 (14.2)	0.091
Diastolic blood pressure, mmHg	79.5 (6.6)	76.7 (10.8)	0.252
HRV, RMSSD	56.2 (24.0)	54.0 (15.9)	0.686
HRV, SDNN	76.0 (19.8)	82.0 (26.5)	0.492
**RECOVERY**			
Systolic blood pressure, mmHg	114.5 (10.6)	109.5 (12.7)	0.103
Diastolic blood pressure, mmHg	70.4 (6.2)	68.1 (12.0)	0.360
HRV, RMSSD	60.1 (18.8)	64.2 (23.1)	0.479
HRV, SDNN	54.2 (18.7)	58.4 (21.7)	0.430
Δ **BASELINE—EXPERIMENT**			
Systolic blood pressure, mmHg	13.7 (7.6)	9.8 (11.2)	0.120
Diastolic blood pressure, mmHg	9.9 (5.3)	6.1 (7.4)	0.028
HRV, RMSSD	−4.9 (16.0)	−15.1 (20.6)	0.044
HRV, SDNN	24.3 (22.1)	17.9 (26.9)	0.331
Δ **EXPERIMENT—RECOVERY**			
Systolic blood pressure, mmHg	−10.1 (6.9)	−9.6 (13.2)	0.844
Diastolic blood pressure, mmHg	−9.1 (5.3)	−8.7 (6.6)	0.795
HRV, RMSSD	4.0 (15.8)	10.2 (15.9)	0.148
HRV, SDNN	−23.5 (16.4)	−23.7 (17.5)	0.966
Δ **BASELINE—RECOVERY**			
Systolic blood pressure, mmHg	3.6 (7.0)	0.2 (11.5)	0.176
Diastolic blood pressure, mmHg	0.9 (5.0)	−2.6 (7.7)	0.049
HRV, RMSSD	−1.0 (10.0)	−4.9 (18.3)	0.321
HRV, SDNN	0.8 (12.9)	−5.7 (20.6)	0.153

*Values shown as mean (SD). Note that due to missing data on some measurement occasion, deltas are not identical to time point differences. Δ, delta; HRV, heart rate variability; mmHg, millimeters of mercury; RMSSD, square root of the mean squared differences of successive normal-to-normal intervals; SDNN, standard deviation of normal-to-normal intervals*.

**Figure 1:**
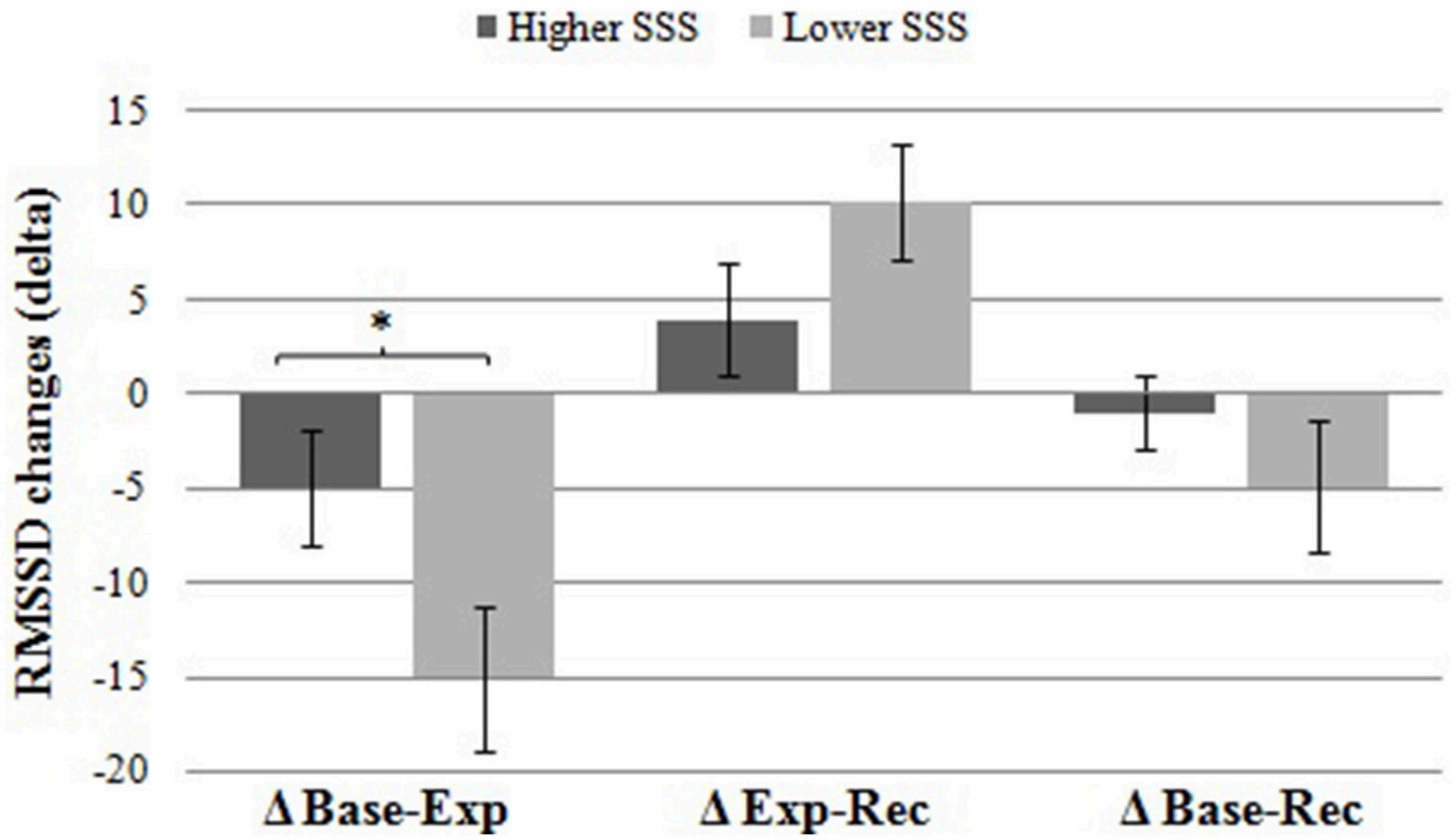
**Differences in HRV (RMSSD) reactivity between groups**. Participants in the low SSS group showed a significantly higher RMSSD decrease (±SE) from baseline to experimental manipulation compared to participants in the high SSS group. There were no significant group differences in change from experimental manipulation to recovery or from baseline to recovery (*p* > 0.1). ^*^p = 0.027.

In a further analysis, we observed whether RMSSD reactivity differed between the low and high SSS groups. For this purpose, changes in RMSSD (delta) were calculated subtracting pre from post values (Table 2). Differences between the two experimental groups were examined using analyses of covariance (ANCOVA). Results indicated that participants in the low SSS condition showed a significantly higher RMSSD reactivity (as reflected by a significantly higher RMSSD decrease), *F*_(1, 49)_ = 5.19, *p* = 0.027, $\eta_{\text{p}}^{2}$ = 0.096, from baseline to experimental manipulation than participants in the higher SSS group. Figure 1 illustrates this effect. There were neither significant group differences in reactivity from experimental manipulation to recovery, nor from baseline to recovery (*p* > 0.1). So, as hypothesized, a social comparison with high-status individuals (low SSS condition) elicits stronger cardiovascular reactivity than a social comparison with low-status individuals (high SSS condition).

The experimental manipulation did not influence blood pressure (*p* > 0.1).

## Discussion

The present study aimed to extend the existing findings on associations between SSS and cardiovascular health by examining whether a short-term experimental manipulation of SSS affects cardiovascular processes. Our main finding is that a temporarily lowered SSS leads to larger HRV (RMSSD) decreases.

This study is the first to show that an experimental manipulation of SSS effectively influences cardiovascular processes. HRV is an indicator of autonomic balance (i.e., dynamic balance of sympathetic and parasympathetic systems). Change in HRV reflects the ability of the autonomic nervous system to adapt to various bodily and environmental demands including respiration, hemodynamic and metabolic processes, sleep and posture changes, physical exercise and mental stressors (Thayer et al., 2012). RMSSD, in particular, reflects short-term alterations of HRV. Decreased RMSSD indicates reduced parasympathetic activity, which has been considered a risk factor for cardiovascular disease (Thayer and Sternberg, 2006; Thayer et al., 2010, 2012). Thus, our findings are in line with previous cross-sectional and longitudinal research suggesting that lower SSS may be related to parasympathetic withdrawal, a potential risk factor for cardiovascular disease (Hegar and Mielck, 2010; Euteneuer, 2014).

Although the specific mechanisms by which SSS may lead to autonomic imbalance are in dire need of further research, one pathway might involve hyperactivity of the hypothalamic-pituitary-adrenal axis causing increased catecholamine release (Euteneuer, 2014). Catecholamines activate the sympathetic nervous system by binding to adrenergic and dopaminergic receptors. Chronically increased catecholamine levels would therefore result in chronic overactivation of the sympathetic nervous system, causing autonomic imbalance. This interpretation is also consistent with findings from a study among healthy adults, indicating that lower SSS predicts reduced *in vivo* responsiveness of the β-adrenergic receptor, a marker for chronically increased catecholamine levels (Euteneuer et al., 2012).

We did not find significant effects of SSS on blood pressure in this study. Although there are some studies suggesting associations between lower SSS and increased blood pressure or even hypertension, there are also some that did not (Hegar and Mielck, 2010). Short-term blood pressure depends on interactions between heart rate, cardiac output, baroreflex, afterload, and arterial compliance. In the long term, blood pressure depends on salt and water balance which are hormonally controlled by the renin-angiotensin-aldosterone system and vasopressin (Chopra et al., 2011). The single short-term manipulation of SSS in our study may not have been sufficient to affect these complex regulatory systems; at least not during the time of measurement. Possibly, more intense or long-term experiences of lower SSS would affect blood pressure. There is evidence from both cross-sectional and prospective studies that chronic autonomic imbalance, as represented by a chronically decreased HRV, may at least partly account for the link between SSS and blood pressure (Thayer et al., 2010). This would support the assumption that increased blood pressure rather reflects long-term effects of SSS which might explain why no differences in blood pressure were obtained in this study.

It is important to recognize that our sample characteristics limit the generalizability of the present findings. Future studies should investigate community based samples to increase validity. A further limitation might be that we did not include a passive control condition without any SSS manipulation. Future studies might want to consider an additional control group. Moreover, future research might also profit from alternative manipulations, for example involving direct experiences of high vs. low social status.

In conclusion, our results suggest that already temporary shifts of one's SSS have measureable effects on cardiovascular processes. They further support the causal role of SSS in the development of cardiovascular disease. A possible mechanism linking low SSS to poor cardiovascular health might be chronic autonomic imbalance as represented by decreased HRV.

## Author contributions

All authors listed, have made substantial, direct and intellectual contribution to the work, and approved it for publication.

### Conflict of interest statement

The authors declare that the research was conducted in the absence of any commercial or financial relationships that could be construed as a potential conflict of interest.
